# Investigation of Carbohydrate Recognition via Computer Simulation

**DOI:** 10.3390/molecules20057700

**Published:** 2015-04-28

**Authors:** Quentin R. Johnson, Richard J. Lindsay, Loukas Petridis, Tongye Shen

**Affiliations:** 1 UT-ORNL Graduate School of Genome Science and Technology, Knoxville, TN 37996, USA; E-Mail: qjohnso3@utk.edu; 2 Department of Biochemistry and Cellular & Molecular Biology, University of Tennessee, Knoxville, TN 37996, USA; E-Mail: rlindsa2@utk.edu; 3 Center for Molecular Biophysics, Oak Ridge National Lab, Oak Ridge, TN 37830, USA; E-Mail: petridisl@ornl.gov

**Keywords:** protein-carbohydrate interaction, binding free energy, molecular dynamics simulation, lectin

## Abstract

Carbohydrate recognition by proteins, such as lectins and other (bio)molecules, can be essential for many biological functions. Recently, interest has arisen due to potential protein and drug design and future bioengineering applications. A quantitative measurement of carbohydrate-protein interaction is thus important for the full characterization of sugar recognition. We focus on the aspect of utilizing computer simulations and biophysical models to evaluate the strength and specificity of carbohydrate recognition in this review. With increasing computational resources, better algorithms and refined modeling parameters, using state-of-the-art supercomputers to calculate the strength of the interaction between molecules has become increasingly mainstream. We review the current state of this technique and its successful applications for studying protein-sugar interactions in recent years.

## 1. Introduction

Carbohydrates are an important class of macromolecules that serve various roles in biological systems [1,2]. Besides the commonly-known functions for metabolic energy (saccharides, such as starch and glycogen, provide fuel for biological systems) and for mechanical support (saccharides, such as cellulose, are the essential building block of cell walls), carbohydrates can play roles in signal transduction, immune response, cell trafficking, cell adhesion and cell-cell interaction. Many of these roles involve protein-carbohydrate interaction and selective protein recognition in the presence of carbohydrates.

The nature of the structure and physical properties of carbohydrates presents unique challenges for designing carbohydrate recognition sites. First of all, at the monosaccharide level, sugar residues are largely similar. There exists a large set of common monosaccharides; many of them are isomers with subtle differences, and they lack distinct chemical groups. Secondly, beyond the monomer level, the potential connection of these monomers via glycosidic bonds is even more complex. Thirdly, glycomics are not directly coded in genomics, unlike proteomics. Thus, the primary structures of sugars (sequence information) are a lot more susceptible to the stochastic nature of chemical reaction events [3]. Such randomness makes the sensing of the consensus of sugar structures difficult. This feature can even be exploited by viruses to evade antibodies [4].

These features of carbohydrates also make the quantitative study of carbohydrate recognition using many traditional methods difficult in several aspects. (1) Impurity: One can imagine that the heterogeneity of primary structures makes purification difficult, which is the necessary first step of any *in vitro* examination. (2) Specificity: The word “recognition” means more than a strong binding affinity between a sugar and the corresponding sensing protein. Strong binding means little if the protein cannot selectively target a specific sugar. Thus, the selectivity of a particular protein-carbohydrate interaction can only be evaluated by examining that protein interaction with large sets of “background noise” carbohydrates (sugars with similar structures to the target). (3) Cooperativity: Since the sugar-protein interactions are largely weak, often multiple binding sites can get involved to enhance the signal. Cooperative binding can be involved in sugar recognition. Cooperativity makes the binding affinity of sites difficult to measure, as the properties of one site depend on the binding status of the others. We argue that these difficulties can be circumvented using the computational study of sugar recognition [5,6,7,8]. Indeed, in the last three decades, there have been extensive applications of computer simulations of biomolecular systems to characterize their biophysical features. Computational methods can be used to directly model a desired sugar species and avoid the purification problem completely. Large-scale parallel computing can also be used to scan a large set of sugar structures and binding scenarios with great efficiency. Furthermore, perturbative binding affinity calculations can provide high-resolution results in a cost-effective fashion. Moving forward, we will review the current progress made in the field of the computational study of protein-sugar interactions, the successes achieved and the challenges faced. Since there are many successful results achieved in this field, this work is not meant to be an extensive review of the whole field. Rather, we focus on raising awareness that computational methods can be a powerful tool in the field of sugar recognition.

With a better understanding of nature’s design of proteins that recognize sugars through experimental and computational studies, we may further enhance our ability to engineer drugs and proteins. For example, oseltamivir and other drugs were designed to block the flu virus [9], while a set of novel proteins block HIV [10]. We may also further design molecular sensors and even pathways involving the desired carbohydrate-protein interactions. Beyond recognition by proteins, the specific interaction between carbohydrates and RNA is another important area that can benefit from the study of sugar recognition [11]. Likely, many of the computational methods developed for studying protein-sugar interaction will also be applicable to the RNA-sugar interaction.

## 2. Structural Features of Carbohydrate-Recognizing Proteins

**Figure 1:**
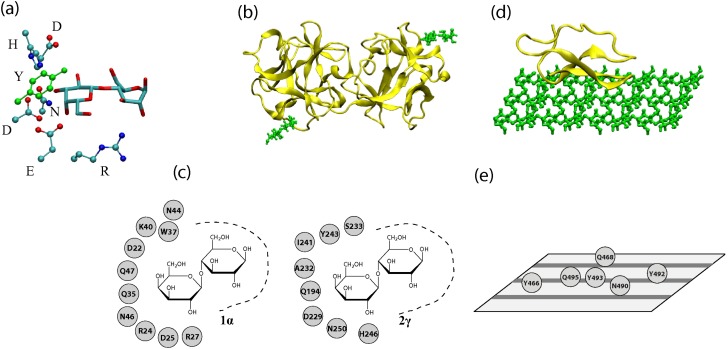
(**a**) A three-dimensional representation of a binding pocket of a lectin (2γ site of the ricin B chain). Only the sugar residues and amino acid residues involved in ring-stacking and hydrogen bonding are shown. Hydrogen and backbone atoms are excluded for clarity. (**b**) A three-dimensional representation of a lectin (ricin chain B, PDB Code 2AAI) with lactoses bound at sites 1α (lower-left) and 2γ (upper-right). (**c**) A depiction of binding sites 1α and 2γ with possible hydrogen bond and ring stacking residues shown. (**d**) A three-dimensional representation of the cellulose binding module (CBM) of a CAZyme (Cel7A with cellulose modeled by M. Crowley’s group) (**e**) Residues of Cel7A’s CBM that interact with cellulose. Cellulose chains are represented by gray stripes on the plane.

Obtaining the structural information of protein-carbohydrate complexes is often the first step for evaluating the strength and specificity of carbohydrate recognition via computer simulations. Since there is a variety of carbohydrates, there are many carbohydrate-binding proteins and carbohydrate-active enzymes that target these sugar molecules. Thus, there are various ways of classifying these interactions. One is to classify the interactions according to the size of the carbohydrate. Some proteins are mono- and oligo-saccharide sensors, while others interact with oligosaccharides and polysaccharides. Another classification is from the protein perspective, whether the protein is purely a binding protein or contains a catalytic module (of the same polypeptide chain). The former group, those that only bind specifically to carbohydrates, are called lectins, while the latter are carbohydrate-active enzymes (CAZymes) that modify the sugars with which they interact. We will review the structural features of the sugar recognition components of both lectins and CAZymes. Both CAZymes and lectins use β-strands in their secondary structure to construct recognition motifs and to design the binding site in general. Examples of sugar-interacting structures from a lectin and a CAZyme are shown in Figure 1. It is interesting to point out that many lectins and CAZymes may share similar folds, such as the β-trefoil.

Lectins are a group of proteins known for their ability to recognize saccharides, but unlike CAZymes, they usually lack enzymatic activity. While lectins have a relatively weak affinity for monosaccharides due to the shallow nature of these binding pockets, they show an increased binding affinity for more complex oligo- and poly-saccharides [12,13]. They are commonly observed as oligomer aggregates, and each lectin monomer typically contains from one to three carbohydrate-recognition domains (CRDs) [12,14]. This quality allows multivalent interaction with complex carbohydrates, contributing to the specificity and the increasing binding strength [13,14]. Lectins can be classified according to the primary and tertiary structural similarities of their CRDs into L-type, C-type, R-type and several others [15,16]. The most common of these, C-type lectins, are characterized by their calcium dependence and a fold, called the C-type lectin-like domain (CTLD) [17]. This motif consists of a double loop joined by two disulfide bridges, with conserved hydrophobic and hydrophilic residues [18]. All CTLDs of carbohydrate-interacting proteins include a calcium ion, which mediates substrate binding [18,19]. R-type lectins contain CRDs with structures similar to the fold of the ricin B chain, a structure called a β-trefoil fold [2,20]. On the other hand, L-type lectin CRDs share a jelly-roll fold, a β-barrel structure formed by multiple pairs of antiparallel β-sheets [2].

Carbohydrate-active enzymes are proteins that break down, construct or otherwise modify carbohydrates [21,22]. In addition to a catalytic module, CAZymes contain carbohydrate-binding modules (CBMs) responsible for substrate recognition. One role of CBMs is to maintain a high concentration of enzyme near the substrate polysaccharide. Keeping the catalytic domain in proximity to its substrate increases the efficiency of the enzyme. Some CBMs have been found to target not only particular glycans, but particular segments of polysaccharide chains. Some CBMs can also serve to disrupt carbohydrate surfaces [22]. Upon binding to a substrate surface, the crystalline organization is disrupted and the surface area of the substrate is increased, making it more accessible to the catalytic domain.

CBMs can be classified in multiple ways. Particularly, they may be classified into three “types” from the carbohydrate perspective. Type A modules interact with insoluble glycan surfaces (such as the surface of cellulose), Type B binds individual glycan chains, and Type C binds small sugars, mono-, di- and tri-saccharides. CBMs can also be classified from the protein perspective in detail. While CBMs show a huge amount of variation in amino acid sequence (70 families), their tertiary structures can be described in terms of just seven “fold families” according to the similarities of their tertiary structure [21,23]. While these fold families share structural motifs, due to varying sequences, it is not always possible to predict specific sugar recognition based on the fold family alone.

The most commonly-observed fold family is the so-called “β-sandwich”, composed of two β-sheets, each with three to six antiparallel β-strands and always containing a metal ion [24]. The next most common fold is the “β-trefoil” fold, which is consistent with that of the R-type of ricin. This fold consists of three subunits with the resultant structure being a β-barrel capped by three hairpin turns, capable of binding up to three carbohydrate molecules [21,24]. While the β-sandwich and β-trefoil fold structures are adaptable to many different substrates, some fold families are more specific. For example, the cellulose-binding and oligosaccharide/oligonucleotide-binding (OB) motifs appear to be specialized for cellulose and chitin recognition. These are small planar CBMs, composed of a β-sheet and coil, whose substrate specificity is mediated primarily by ring interactions with aromatic amino acid residues. Another example is the hevein fold family, comprised of mostly a coil with two small β-sheets and a short helical region and specific to chitin. A variation of the hevein fold with an additional β-sheet is found in one CBM family and makes up another fold family [21].

All of these classifications of protein-sugar interactions come from the collections of the structural information of the complexes. Databases exist to help classify carbohydrate-binding proteins. These databases and associated tools also serve as the starting point of computer simulation and modeling [25,26,27,28,29]. For example, the Glyco3D database is comprised of six databases containing three-dimensional structures and other information for glycans and proteins that interact with them, including lectins, glycosaminoglycan binding proteins, monoclonal antibodies with carbohydrate ligands and glycosyltransferases [26]. The Carbohydrate-Active Enzymes database (CAZy) contains present knowledge of experimentally-characterized CAZymes [23]. General algorithms exist to classify three-dimensional carbohydrate structures in PDBs and to describe how they interact with the protein environment [27]. These structural databases can provide valuable bioinformatics on protein-carbohydrate interaction and can serve as the starting point of high-resolution computer simulation study of carbohydrate recognition.

## 3. Physical Interactions at the Recognition Sites

The actual recognition by proteins is realized by the complicated, but direct physical interactions between the protein and carbohydrate [30]. There are five types of physical interactions often involved in recognition: (1) hydrogen bonding; (2) sugar ring-aromatic ring stacking; (3) salt bridge interaction of charged carbohydrate moieties; (4) metal ion-mediated interaction; and (5) solvent-mediated interaction.

Hydrogen bonding is the most important interaction between carbohydrates and proteins. Each carbohydrate residue generally has multiple hydroxyl groups and a ring oxygen. These chemical groups act as hydrogen donors and/or acceptors. Often, the recognition site of the protein has either the side chains of polar amino acid residues or protein backbone components that form hydrogen bonds with the carbohydrate. Since most hydrogen bonds formed are of the type O-H. . .O or N-H. . .O, the strength of the typical H-bond interaction involved in recognition is about 5–6 kcal/mol in a vacuum and 1.5–2 kcal/mol in aqueous solution [31,32].

Besides the well-studied hydrogen bonding, another important, but yet to be further explored, interaction is the stacking interaction [30,33,34] between the pyranose ring of carbohydrates and aromatic amino acids, Trp, Phe and Tyr. Having a similar nature to that of ring-ring stacking interactions (*π* *− π* bond) between the base pairs of DNA, this sugar ring-aromatic ring interaction (CH*−π* bond) has been reported to be essential for the recognition of many monosaccharides. Through quantum chemistry calculations, this ring-ring interaction has been reported to be about 3–6 kcal/mol in the gas phase [33]. However, the experimental estimation of this interaction in solution is much weaker, reported to be about 0.5–0.8 kcal /mol [34].

A third type of physical interaction is direct Coulombic interaction. Although most sugar residues are neutral, a few important monosaccharides are negatively charged, such as the sialic acid family [1,2,35]. In such cases, the electrostatic interactions might be crucial for recognition. For example, positively-charged arginine is known to be essential for a group of sialic acid-recognizing lectins [36,37,38]. Thus, the salt-bridge interactions were suspected to play an important role in the recognition of certain sugars. However, since conservative mutation of Arg to Lys can abolish the binding affinity of a subset of these lectins, this indicates that interactions other than electrostatic could be more important for those specific cases [39].

Metal ions, such as calcium, can also play a role in protein-carbohydrate interaction [18]. There is a class of ion-mediated carbohydrate-protein interactions. Besides stabilizing the recognition site of the protein and, thus, indirectly enhancing the interaction, metal ions can form direct contacts with both the protein and sugar [18,40]. Besides ions explicitly coordinating the interactions, the solvation effect can also play a role in carbohydrate-protein binding. Although saccharides in general are largely hydrophilic, one would believe that desolvation effects play a smaller role in carbohydrate-protein interaction compared to that in protein-protein interaction [41]. Nevertheless, desolvation is important for some carbohydrate-binding proteins and their target polysaccharides. One well-known example is the interaction between the hydrophobic face of cellulose and the CBM of cellulase [42,43,44].

## 4. Computer Simulation of Protein-Carbohydrate Complexes

Computer simulation has become an important tool in molecular biology in general, since most research subjects in this field are microscopic and sensitive to environmental perturbation, which can make direct observation difficult [45]. The development of *in silico* methods [46] can provide a complementary approach to traditional approaches, such as nuclear magnetic resonance, crystallography and scattering techniques [47,48]. Atomistic simulation methods, after careful validation by comparison to experiment, are unmatched in efficiently presenting detailed and direct quantitative information with nanoscale resolution [49,50,51]. These simulation techniques directly track the three-dimensional movement of all of the atoms of the molecular complex according to physical laws and have been successfully applied to study the structure, dynamics and thermodynamics of biomolecules [52]. Simulations require two types of input information: (1) the initial structure of the system, which can be obtained from experimental data or modeling; and (2) the so-called “force field”, empirical potential energy functions (derived from quantum chemistry calculations and/or fitted from experimental observations) that determine how atoms interact. Based on this information, a computer simulation can be performed for a complicated biomolecular system using supercomputers. The evolution of structures of the systems are recorded as time-stamped snapshots (trajectories) for analysis.

The early biological applications of atomistic molecular dynamics (MD) simulation focused on proteins, with the first simulation performed on the protein BPTI [53] almost 40 years ago. Thus, the force-fields of proteins are quite mature. Later, the application of simulations was expanded to include other important biomolecules, such as nucleic acids, lipids and carbohydrates, and corresponding force fields have been developed [54]. Particularly, carbohydrate force fields at all-atom resolutions, such as CHARMM [55,56,57], GLYCAM [5,58], GROMOS [59] and OPLS [60], have been used to study the behavior of polysaccharides [61,62]. MARTINI-based coarse-grained force fields, which provide a useful methodology to study large systems on long time scales, have also been developed for polysaccharides [63,64]. Besides the generic advanced methods used in sampling and free energy calculation that we will discuss below, advanced simulation algorithms that target the features of carbohydrate force fields have been developed, as well [51,65]. Indeed, the dramatic energy landscape of torsional angles, unique puckered ring conformations of carbohydrates and many other factors make the sampling of carbohydrates demanding [66]. These enhanced sampling methods ease the difficulty of crossing over high energy states of carbohydrates.

There are still several challenges for carbohydrate force field development. Polysaccharides, with their abundant hydroxyl groups, are known to form various intra- and inter-molecular hydrogen bonds. For example, modeling of the highly directional hydrogen bonds of polysaccharides in a crystal environment poses significant challenges and is hypothesized to lead to overestimation of carbohydrate crystal volumes [57,67]. In contrast, proteins have a well-defined secondary structural arrangement of hydrogen bonding, and nucleic acid hydrogen bonds can be easily tracked by base pairing. Furthermore, sugar residues are flexible biomolecules that adopt multiple stereoisomers, and care must be taken to optimize force fields that accurately treat their inherent conformational complexity [5,55]. For example, six-membered pyranose rings are puckered and adopt stable chair or boat conformations. Furthermore, when covalently linked to form polysaccharides, monosaccharides are linked with various types of glycosidic linkages, each of which can exist in either an α or β anomer [56,68]. Accurately quantifying the conformational properties of oligosaccharides based on the values of their glycosidic torsion angles is essential when predicting carbohydrate-protein structures [68].

Another potential challenge is a classical description of biomolecules and ions. For one, the calcium ions and metallic bonds in MD simulations [69] are traditionally difficult. Clearly, this may affect the small class of metal ion-mediated protein-sugar interactions. This is a general issue of metal ions in simulations and is not specific to our current topic. Furthermore, simulation of classic force fields provides fast calculations by only tracking the motions of nuclei. However, there are certain effects that cannot be described using such force fields. One well-known example is the cooperativity of hydrogen bonds [70]. Indeed, polarizable force fields may be necessary to accurately describe carbohydrate recognition and have been developed to incorporate such many-body interactions in carbohydrate simulations [71,72,73].

A further technical challenge exists when combining atomistic carbohydrate and protein force fields, which arises from inconsistency with the treatment of 1–4interactions. Empirical force fields normally disregard the 1–2 (between two covalently bonded atoms) and 1–3 (between two atoms separated by two covalent bonds) nonbonded interactions. However, 1–4 nonbonded interactions (atom separated by three covalent bonds) are considered in addition to internal parameters (dihedral term) in the determination of conformational energies. A 1–4 scaling factor of the non-bonded term is used to avoid potential exaggeration of short-range repulsion caused by the 6–12 Lennard–Jones interaction potential. For example, the AMBER [74] force field for proteins has chosen the scaling factor to be 1/1.2, whereas CHARMM force fields [75] do not scale this interaction, *i.e.*, the scaling factor is 1.0. Most carbohydrate force fields also do not scale the 1–4 interactions [5,59,75]. However, the OPLS carbohydrate force field includes 1–5 and 1–6 interaction scaling to improve the agreement with experiments [60]. In practical terms, in a simulation of a protein-carbohydrate non-covalent complex, one can simply keep a different 1–4 scaling factor for the protein and for the carbohydrate. However, in a simulation of a glycoprotein, in which a carbohydrate is covalently bonded to a protein, it is difficult to keep two separate scaling factors [76]. Nevertheless, simulations of glycosylated proteins often provide insight into the intramolecular interactions between amino acid and carbohydrate residues [77,78,79,80].

Despite these challenges, simulations of carbohydrates, glycoproteins and protein-carbohydrate complexes are helping researchers understand the mechanisms of carbohydrate recognition in various systems [8,66,81,82,83,84].

## 5. Free Energy Calculation of Protein-Carbohydrate Interactions

With the quantitative spatial data obtained by molecular dynamics simulations in hand, analytical methods are required to reveal the binding mechanism between protein and carbohydrate. As stated in the Introduction, ultimately, the recognition ability is measured as the selective and strong binding between the recognition site and the target carbohydrate. Thus, an important quantitative property that determines the strength of binding is free energy [85]. Although quantitative spatial data can be obtained directly by molecular dynamics simulations, further analysis methods are often required to obtain the thermodynamics of carbohydrate binding.

In general, the binding thermodynamics between two (bio)molecules in solution can be loosely divided into four terms: (1) a direct enthalpic term of binding energy, which is described directly by molecular mechanics (MM); (2) an entropic term due to the loss of overall translational and rotational degrees of freedom (DOFs) upon binding [85]; (3) an entropic term due to the change of the internal degrees of freedom [86]; and (4) a solvent term, including desolvation effects (such as the solvent accessible (SA) method [87]) of the binding and the polar solvent effects (such as Poisson–Boltzmann (PB) or generalized Born (GB) methods [88,89,90]). The overall rotational DOFs can be coupled with internal DOFs, and the clear separation for large and flexible molecules is difficult.

There are a large number of methods that derive free energy changes (∆*G* = *G_cplx_−G_protein_−G_sugar_*) based on information obtained directly from simulation results. In this work, we do not intend to provide a comprehensive review on free energy calculation and the thermodynamics of binding in general. Rather, we present an overview of this subject with the focus on applications to carbohydrate-protein systems. Roughly speaking, two groups of free energy methods are commonly applied for studying sugar recognition. One group of methods, including the widely applied MM-PBSA (“MM” + “PB” + “SA”) and MM-GBSA methods [41,87], focuses on the “end-point” calculations and can obtain the relative binding free energy. They often ignore the effect of the concentrations of sugar and protein. Thus, these methods do not provide the absolute binding free energy. However, they are demonstrated to be suitable for the calculation of binding free energy of relatively large carbohydrate systems (beyond mono- and di-saccharides). On the other hand, the second group of methods, including the well-known double decoupling method [91,92] and other free energy perturbation/thermodynamics integration methods [52,93], applies rigorous statistical mechanics and can obtain the absolute binding free energy or accurate free energy changes between different states. This group of “alchemical transformation” methods is more suitable for the recognition of small sugars, as the “path” calculation for larger systems can be more computationally intensive. Thus, the two groups of methods can complement one another.

As it stands, there is no universally efficient method for free energy calculation for all types of systems; the suitable method depends on the nature of the question and the resolution of the answer desired. Therefore, depending on the size of the sugar and whether the absolute binding strength or just a relative binding free energy (specificity) is desired, different methods can be selected. For example, Nyholm *et al*. successfully estimated the binding energies of hevein with a mono- and di-saccharide using the double decoupling method [94]. Their simulations correctly predict the enthalpy-dominant nature of the hevein-carbohydrate interactions and correctly estimate the binding free energy of the hevein-GlcNAc complex as −2.0 kcal/mol (experimental value −2.0 kcal/mol); however, they overestimated the hevein-(GlcNAc)2 as −5.2 kcal/mol (experimental value of −3.8 kcal/mol). On the other hand, many studies did not provide the accurate absolute values of binding, but provide the relative correct ranking. For example, Naismith *et al*. performed nanosecond-scale molecular dynamics simulations followed by MM-PBSA analysis to explain why a pentasaccharide binds to concanavalin a (lectin) with the same affinity as a trimannoside [95]. They found that MM-PBSA correctly ranks the free energies of binding of a set of protein-carbohydrate complexes [95]. Liang *et al*. studied the different binding free energies of mannose and glucose to the C-type lectin rat mannose binding protein [96]. Their result was qualitatively accurate, but overestimated the experimentally-determined relative binding free energy by a factor of two [96]. Obtaining a correct ranking order, but overestimating the strength of the binding by about a couple fold seems to be a common occurrence in many simulation studies. Pathiaseril and Woods modeled the binding free energies of several mono- and tri-saccharides with monoclonal anti-Salmonella antibody Se155-4 relative to the affinity of the wild-type carbohydrate ligand [7]. The simulations were shown to reasonably reproduce the known geometries of the ligands and the ligand-protein complexes [7]. The predicted values agree closely with experimental values; the difference is less than 1 kcal/mol in most cases. Typically, the error bars of measurement are about 0.2 to 1.0 kcal/mol for both simulation and experimental sides. Kuntz *et al.* explored how structurally similar compounds yield vastly different affinities with neuraminidase [97]. They found that improved positioning of ligand atoms in the active site due to polar and hydrophobic functionalities may be a major factor for increasing ligand selectivity. They also found that more potent ligands make less use of water-mediated interactions in the active site [97]. Though these studies have different focuses, they all provided new insights on sugar recognition by proteins. There is a vast variety of potential applications and carbohydrate recognition systems ready for researchers to perform computer simulation studies. With more systems being studied, the methods used will be further calibrated and automated. They will provide better simulation and calculation ability as carbohydrate, protein and solvent force fields are refined and simulation and analysis algorithms are improved.

## 6. Cross-Talk Between Recognition Sites

As stated, carbohydrate-interacting proteins, such as lectins, are known to have multiple recognition sites. One can speculate the advantage of such designs, a simple answer is that multiple binding sites increase the chances of binding and, thus, strengthen the interaction. Besides the simple additive effects of having multiple binding sites, the concept of cooperative (allosteric) binding has been raised in various sensing systems [98,99,100,101]. The so-called allosteric binding refers to the concept that the binding strength of one binding site can be positively (or negatively) influenced by the binding status of another distant binding site. Indeed, it is well known that such a cooperative effect can make the signal sharper, *i.e.*, the dosage-response curves show a sigmoidal shape and have a shorter transition area between the “on” and “off” states. This is especially important for the sugar-lectin-type of interaction, as the binding affinity is usually relatively weak at 2–4 kcal/mol [102].

Despite its importance in sensing and signaling systems, allostery has not been emphasized by traditional methods. There are two potential challenges in detecting the level of allostery. For one, researchers traditionally judge whether there is allostery by determining whether there are two conformations, such as the R versus T states of proteins. However, capturing two or even more different conformations can be difficult, as the structural changes that most allosteric effects involve are quite subtle. Furthermore, the ensemble view of allosteric changes was only presented in recent years, which emphasizes that fluctuation effects alone can induce allosteric changes without the need to alter the ground state or mean structure level [103]. Another problem is model (parameter) fitting. For example, for a simple two-binding site system, researchers often assume one of the two following scenarios to be correct: (1) two binding sites have intrinsically identical binding properties, but have a (positive or negative) cooperative binding between them; or (2) the two binding events are independent with respect to each other, and one site is intrinsically stronger than the other. Both Model (1) and (2) require only two parameters [104,105]. However, a more complete model is that two sites have intrinsically different binding strengths, but also have a certain level of cooperativity. Thus, a total of four parameters are required to fully characterize such situations. Often, traditional models opt to fit a simple model with fewer parameters due to the limited availability of data, while computational methods can calculate the binding free energy of various combinations of binding states [106].

**Figure 2:**
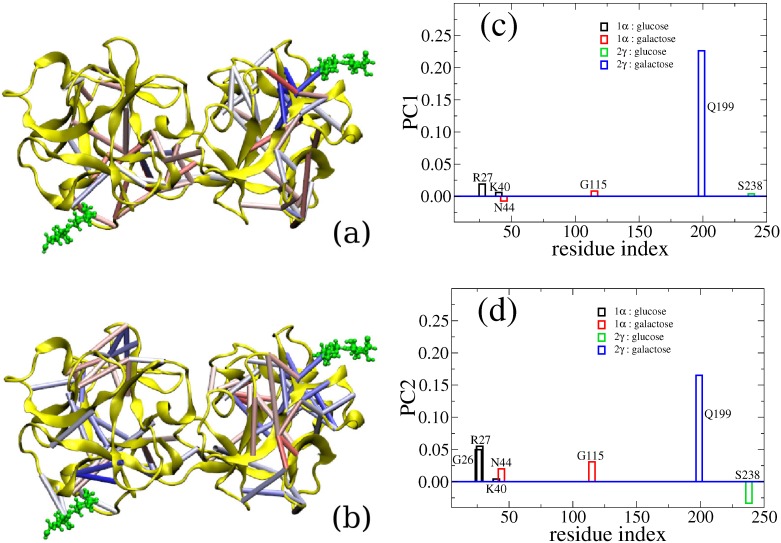
Three-dimensional representations of PC1 and PC2 values are shown in (**a**) and (**b**), respectively. For clarity, only those contacts with absolute values of >0.05 are explicitly displayed as colored cylinders. Elements in eigenvectors PC1 and PC2 that contain the contact interaction between ligands and the protein complex are show in (**c**) and (**d**), respectively.

Here, we use the binding between a lectin (the chain B of ricin) and lactose (the terminal disaccharide of a cell-surface polysaccharide) as an example to illustrate how computer simulation is able to capture the cooperativity between different binding sites. The ricin toxin B chain folds into two β-trefoil structures and contains potentially six sugar recognition sites. Practically, only two of them, sites 1α and 2γ, have retained binding ability. There have been contradictory experimental observations regarding the binding of these two sites, as summarized in our previous report [107]. Thus, we studied this protein-carbohydrate complex through all-atom simulation. We calculated binding free energy between the ricin B chain (RTB) and lactose using the MM-GBSA method and reported that specific residues interact with the saccharide via hydrogen bonding [106]. However, beyond the standard free energy calculation, we also found that there is a level of positive allosteric effect between these two binding sites. Indeed, when both ligands are bound, the interactions between carbohydrates and RTB are stronger than the case with only one ligand bound. To go beyond qualitatively reporting the allosteric binding and to reveal the actual allosteric interactions between the two binding sites, we have analyzed our early simulation data with a new method called CAMERRA (computation of allosteric mechanism by evaluating residue-residue associations), which we have previously applied to study another allosteric system, the nuclear receptor complex of RXR:TR [107]. As displayed in Figure 2, we show the positive cooperativity between the binding events of lactose to sites 1α and 2γ via CAMERRA. We displayed the top two principal components, which can be viewed as a vibrational mode for residue-residue contacts. Here, contact dynamics (breaking and forming of the interactions between amino acid residues) are shown. A positive value indicates contact forming, while a negative value indicates breaking. One can see that PC1 does not show much cross talk between the two binding sites, while PC2 shows overall positive allostery between the contact events, *i.e.*, when lactose increases contact with site 1α, the other lactose increases the contact with site 2γ. Particularly, we found that the interaction between Gln199 and lactose at 2γ was correlated with stronger binding at site 1α by residues Arg27, Asn44, Gly26 and Gly115. The network of residues involved in the crosstalk between the binding sites is visualized with colored cylinders between amino acid residues. Future engineering and design of lectins with multiple binding sites may explore such cross-talk features and enhance the ability of sugar recognition.

## 7. Conclusions

In this work, we reviewed the common schemes used by proteins for carbohydrate recognition and the potential challenges faced in studying the recognition mechanism. We presented how computational methods can assist in the quantification of sugar recognition and, especially, how those methods can circumvent the traditional problems (purification, specificity, and cooperativity) faced. Furthermore, different methods used to calculate the binding free energy between protein and carbohydrate were compared. Successful examples of a binding study via computer simulation were given to demonstrate that this has become a mature technique, despite the existing imperfections, which are being improved.
